# Thermochromism of Highly Luminescent Photopolymer Flexible Films Based On Eu (III) Salts Confined in Polysulfone

**DOI:** 10.3390/ma13235394

**Published:** 2020-11-27

**Authors:** Mani Outis, João Paulo Leal, Maria Helena Casimiro, Bernardo Monteiro, Cláudia Cristina Lage Pereira

**Affiliations:** 1 LAQV-REQUIMTE, Departamento de Química, Universidade Nova de Lisboa, 2829-516 Caparica, Portugal; m.hosseinzadeh@campus.fct.unl.pt; 2 Centro de Química Estrutural (CQE), DECN, Instituto Superior Técnico, Universidade de Lisboa, Estrada Nacional 10, 2695-066 Bobadela, Portugal; jpleal@ctn.tecnico.ulisboa.pt; 3 Centro de Ciências e Tecnologias Nucleares (C2TN), Instituto Superior Técnico, Universidade de Lisboa, Estrada Nacional 10, 2695-066 Bobadela, Portugal; casimiro@ctn.tecnico.ulisboa.pt; 4 Centro de Química Estrutural (CQE), DEQ, Instituto Superior Técnico, Universidade de Lisboa, Estrada Nacional 10, 2695-066 Bobadela, Portugal

**Keywords:** thermal behavior, lanthanide, β-diketonate, polysulfone, polymer films, europium (III), hybrid materials

## Abstract

Here we discuss the influence of two different cations on the emissive properties of the highly emissive [Eu(fod)_4_]^−^ anion. The studied Eu(III) salts were [C_16_Pyr][Eu(fod)_4_] (**1**), and the previously reported [Chol][Eu(fod)_4_]. C_16_Pyr stands for N-cetylpyridinium, Chol for cholinium and fod for 1,1,1,2,2,3,3-heptafluoro-7,7-dimethyloctane-4,6-dionate. **1** is classified as ionic liquid, with melting point close to 60 °C, and presented a luminescence quantum yield of (ϕ) 100%. Ultrabright emissive photopolymers were obtained for the first time using polysulfone as the host matrix. The films were prepared with incorporation of 10% (*w*/*w*) of **1** and **[Chol][Eu(fod)_4_]** in the polymeric matrix, which improved its thermal stability. Additionally, the luminescence of **CholEu(fod)_4_/PSU** presented a strong temperature dependence with a ratiometric thermal behavior.

## 1. Introduction

Carlos and co-workers reviewed recently and exhaustively the luminescence thermometry of lanthanide compounds applied as thermal probes for temperature determination in numerous biomedical implementations [1,2]. This research field has been widely explored in the last decade because of the enormous potential of luminescence thermometry particularly in nanotechnology and nanomedicine [3,4].

The determination of temperature in living cells is of paramount importance, as cancer cells are known for having higher temperatures than those of normal tissues due to the increased metabolic activity [5]. Among others, this is an example where conventional thermometry is not helpful, as this demands precise thermometry down to the nanoscale and the common thermocouples and thermistors are inappropriate below 10 μm [2].

In Eu (III) doped systems, the ratio of the emission intensity between ^5^D_1_→^7^F_J_ and one of the emission bands from the ^5^D_0_→^7^F_1_ or ^5^D_0_→^7^F_2_ transitions has been used as a possible strategy for temperature sensing [6,7,8,9,10,11,12,13].

Here we describe a new approach that will allow the design of new ratiometric temperature sensors based on the highly emissive Eu (III) tetrakis-betadiketonate complexes and the correspondent hybrid materials using as host polysulfone, PSU, as the polymeric matrix. A temperature-dependent degree of asymmetry of the confined Eu (III) complex allows a remarkable modification in the intensity of the hypersensitive transition ^5^D_0_→^7^F_2_, that reflects the temperature to which the complex is exposed.

The nature of the cations in [Eu(β-diketonate)_4_]^−^-based salts can have a strong influence on the structure of the anion and consequently on the optical properties of the complexes [14,15].

Beside the here presented β-diketonate Eu (III) complexes, other important group of lanthanide complexes incorporated into different hosts are those based on the carboxylate ligands. In the case of PEGDMA, poly(ethylene glycol dimethacrylate) hybrids, it was observed a decrease in quantum yield efficiency, Φ, after Eu (III) complex incorporation which may result from the stronger interaction of high energy C–H and O–H oscillators, which significantly quenches the emission of Eu(III) [16].

Developments in polydopamine fluorescent nanomaterials have been recently reviewed by Wang and Li due to their unique physicochemical and biological properties. [17]

Other organic–inorganic hybrid materials, based on silica gel functionalized with DAPTS ([3-(2-aminoethylamino)propyl]trimethoxysilane), APTS ((3-aminopropyl)trimethoxysilane) and TAPTS (3-[2-(2-aminoethylamino)ethylaminopropyl]trimethoxysilane) and containing covalently linked Ln-EDTA complexes of Gd (III), Eu (III) and Tb (III), exhibited intense luminescence when excited between 280–370 nm. The luminescent brightness however is dependent of the combination of the β-diketonate ligand used as antenna and the Ln (III) ion coordinated to the EDTA ligand on the silica gel surfaces. For the Eu (III) ion, the brightest materials are those which contain tta (thenoyltrifluoroacetonate) and dbm (dibenzoylmethanoate) as the luminescence sensitizers, while for Tb (III) the brightest systems were found when bzac (benzoylacetonate) and acac (acetylacetonate) were used as ligands [18]. 

Polysulfones, also called polyaryl sulfones, are a class of polymers with high thermal, oxidative and hydrolytic stability. They are amorphous, transparent thermoplastics that can be molded, extruded, or thermoformed into a wide variety of shapes. Based on this feature, we proposed a new method for evaluating the thermo-sensitivity of Eu-based flexible films by using a ratiometric signal between the electric dipole transition band ^5^D_0_→^7^F_2_ and the magnetic dipole transition ^5^D_0_→^7^F_1_ for **1/PSU** and **CholEu(fod)_4_/PSU**.

We will discuss how changes in the site symmetry of the Eu (III) complexes confined in PSU can be ratiometrically related with temperature to which the complex is exposed. 

## 2. Materials and Methods

### 2.1. Synthesis

Reagent grade chemicals including Polysulfone (beads, average M_n_ ~22,000 by MO, ref.: 182443), were obtained from Aldrich and used without further purification. All solvents for spectroscopic studies were of spectroscopic grade (Aldrich) and used without further treatment.

**[C_16_Pyr][Eu(fod)_4_]** (**1**): 4.1 equivalents (eq.) of NaOH (0.090 g, 1.119 mmol, 50% *w*/*w* aqueous solution) were added to an ethanol solution of 4.1 eq. of Hfod (0.331 g, 1.119 mmol) and left stirring for 2 h. 1 eq. of EuCl_3_·6H_2_O (0.100 g, 0.273 mmol) was then added to the reaction mixture and the resulting solution left stirring for 2 h followed by the addition 1 eq. of N-cetylpyridinium chloride monohydrate (0.098 g, 0.273 mmol). After 1h the solvent was removed under low pressure. To remove the NaCl, the solid obtained was redissolved in CH_2_Cl_2_ and the salt was removed by filtration. The pure compound was obtained as a powder after solvent evaporation, at room temperature with a yield of ca. 80%. Calculated Analysis (Anal. Calcd.) for [C_21_H_38_N][Eu(C_10_H_10_O_2_F_7_)_4_]: C, 44.75; H, 4.80; N, 0.86%. Experimental; C, 44.69; H, 4.91; N, 0.81%. ^1^H-NMR δ (ppm): 15.29 (s, ^4^H-C_16_Pyr), 10.68 (s, ^2,6^H-C_16_Pyr), 7.29 (s, ^3,5^H-C_16_Pyr), 5.29 (s H_α_ fod), 3.29 (t, α-CH_2_-C_16_Pyr), 2.39 (t, β-CH_2_-C_16_Pyr), 1.96 (t, γ-CH_2_-C_16_Pyr), 1.79 (m, CH_2_-C_16_Pyr), 1.35 (m, CH_2_-C_16_Pyr), 0.88 (t, CH_3_-C_16_Pyr). ESI-MS (m/z): 304 [C_21_H_38_N]^+^ (Figure S1), 1333 [Eu(C_10_H_10_O_2_F_7_)_4_]^−^ (Figure S2).

**[Chol][Eu(fod)_4_]:** Equal to **1** but using 1 eq. of CholCl (0.038 g, 0.273 mmol). Anal. Calcd. for [C_5_H_14_NO][Eu(C_10_H_10_O_2_F_7_)_4_]: C, 36.67; H, 3.70%. Experimental; C, 36.61; H, 3.98. ^1^H-NMR δ (ppm): 5.51 (s H_α_ fod), 4.03 (m, -CH_2_ Chol), 3.52 (m, -CH_2_ Chol), 3.26 (s, -CH_3_ Chol), 0.7 (s, -CH_3_ fod).

[C_16_Pyr][Eu(fod)_4_]/polysulfone (**1/PSU**): Polysulfone (0.0568 g) and [C_16_Pyr][Eu(fod)_4_] (0.0057 g) were dissolved with stirring in a closed vial with 5 mL CH_2_Cl_2_. The doped membrane was obtained by leaving the resulting solution evaporating overnight in a loosely closed petri dish at a controlled temperature of 21 °C. **1/PSU** membrane was collected from the petri dish and used without any further treatment. Thickness: 0.01 mm.

**[Chol][Eu(fod)_4_]/polysulfone**: Equal to **1/PSU** but using 0.0593 g of polysulfone and 0.0059 g of [Chol][Eu(fod)_4_]. Thickness: 0.01 mm.

### 2.2. Methods

Microanalyses for C and H were carried using a Thermo Finnigan-CE Instruments Flash EA 1112 CHNS series. FT-IR spectra (range 4000–400 cm^−1^) of the europium salts were collected as KBr pellets (FT-IR grade, Sigma-Aldrich, St. Louis, MO, USA) averaging 64 scans with a maximum resolution of 4 cm^−1^ using a Thermo Scientific Nicolet iS50 FT-IR spectrometer (Thermo Scientific, Waltham, MA, USA). Analysis of the heated sample was performed by heating previously a KBr pellet containing 1 or 2 in an oven. The membranes, **1/PSU** and **[Chol][Eu(fod)_4_]/PSU**, were collected using the same conditions as the Eu-salts but in ATR mode averaging 8 scans.

Spectrometry (ESI-MS). ESI-MS was performed using a Bruker HCT quadrupole ion trap mass spectrometer (Bruker, Bremen, Germany). Sample solutions (~10^−5^ M, acetonitrile) were injected to the ESI source at a flow rate of 150 mL·min^−1^. The capillary temperature was set to 250 °C and N_2_ (cover gas) to a flow rate of 2 L·min^−1^. Both positive and negative modes were used.

The thickness of the membranes was measured on several points of strips of the prepared membranes with a Mitutoyo thickness gauge meter (Mitutoyo, Kawasaki, Japan) with a 0.01 mm.

TGA curves were obtained with samples (~8 mg) in Aluminum crucibles using a thermogravimetric analyzer TA Instruments Q500 (TA Instruments, New Castle, PA, USA), with a heating rate of 10 °C·min^−^^1^.

Luminescence spectra were measured in a SPEX Fluorolog-3 Model FL3-22 spectrofluorimeter using 1 nm slits. The excitation wavelength varied between 344 and 354 nm. Lifetime measurements were run on a LKS.60 ns laser photolysis spectrometer from Applied Photophysics, with a Brilliant Q-Switch Nd:YAG laser from Quantel, using the second harmonic (λ_exc_ = 355 nm, laser pulse half-width equal to 6 ns). Luminescence quantum efficiencies were measured by the absolute method with an Integrated Sphere. All spectra were corrected with correction functions provided by the supplier following standard procedures. For the luminescence quantum yield determination we used equations 8 and 9 of Ref. [19].

Luminescence decays were studied using a LS45-Perkin-Elmer spectrofluorometer in a time-drive mode using a pulsed xenon lamp, performing 2 measurements to confirm the repeatability.

Emission decays were preformed setting the spectral resolution for 2 nm, using a perpendicular geometry relative to the laser excitation and averaging 2–10 measurements for each emission wavelength according to the emission intensity of the sample. Luminescence decay traces for each wavelength were analyzed using least-squares fittings of the experimental data, using Solver from Microsoft Excel. Luminescence studies with variable temperature was performed using an optical fiber focused in the sample that was heated on a thermostatized heating plate (Argolab, Capri, Italy), over a temperature range from 25 to 110 °C. 

## 3. Results

Eu(III), as most lanthanides, have electronic excited states which radiatively decay to the ground state. The most studied lanthanides, in terms of luminescence, are Eu (III), with red luminescence, and Tb (III) with green luminescence, whose excited states are ^5^D_0_ (580 nm) and ^5^D_4_ (490 nm), respectively. Generally, for a higher degree of symmetry around Eu(III) center, the simpler is the emission spectrum, due to the simpler line splitting [20,21].

### 3.1. Luminescence of [Eu(fod)_4_]^−^ as a Function of the Counter-Cation and Temperature in the Solid State 

**Figure 1:**
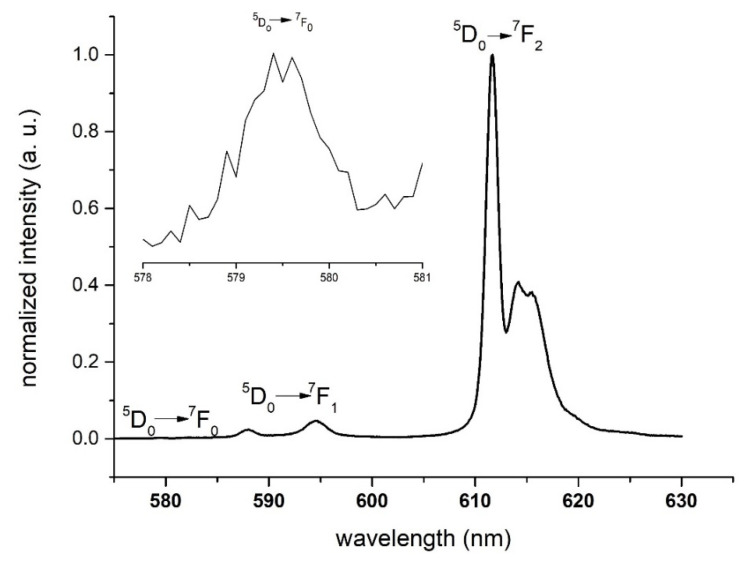
Normalized emission spectra of **1** (black line) relative to the most intense ^5^D_0_→^7^F_2_ transition band at 25 °C in the solid state with λ_exc._ = 345 nm. Inset magnification of ^5^D_0_→^7^F_0_ transition band (wavelengh range 578–581 nm).

The excitation spectrum of **1** was monitored within the Eu(III) ^5^D_0_→^7^F_2_ transition maxima and was completely dominated by a broad band ranging in the UV (ca. 320–400 nm) with maximum of 320 nm (Figure 1). This band is attributed to ligand-centered (S_1_→S_0_) transitions of the fod-diketonate ligand. 

The emission spectrum was recorded with the excitation wavelength set at 320 nm. The transitions to the ^7^F_5_ and ^7^F_6_ levels were not observed due to limitation of the detection capacity of the spectrofluorimeter.

Figure 1 presents the most intense transitions observed for **1** and shows two electric dipole (ED) transitions; the ^5^D_0_→^7^F_0_, with very low intensity and the ^5^D_0_→^7^F_2_ transition very often the most intense band of the luminescence spectra. The ^5^D_0_→^7^F_1_ transition is magnetic dipole dependent and its intensity is largely independent of the environment and can be considered in a first approximation to be constant. For this reason it is usually used to calibrate the intensity of the Eu(III) luminescence spectra [22]. It is not uncommon that the ^5^D_0_→^7^F_2_ transition is 10 times more intense than the ^5^D_0_→^7^F_1_ transition in Eu(III)-β-diketonate complexes.

#### 3.1.1. Cation Effect

**Scheme 1:**
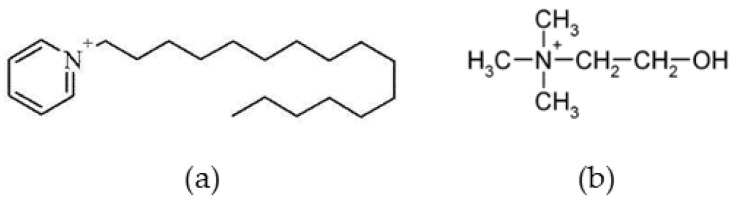
(**a**) N-cetylpyridinium and (**b**) Cholinium.

The cation effect on the [Eu(fod)_4_]^−^ emission properties to be discussed here are N-cetylpyridinium ([C_16_Pyr]^+^, Scheme 1a) and Cholinium ([Chol]^+^, Scheme 1b) 

For the [P_6,6,6,14_][Eu(fod)_4_] ionic liquid, a gradual color change was observed from light yellow to a deep red/purple around 80 °C. This was attributed to partial β-diketonate decomplexation, with concomitant establishment of a strong anion-cation interaction [14]. This color change, induced by heating and previously reported also for [Chol][Eu(fod)_4_], was not observed for **1**, that maintained its light-yellow color unaltered until melting at temperature close to 60 °C.

**Figure 2:**
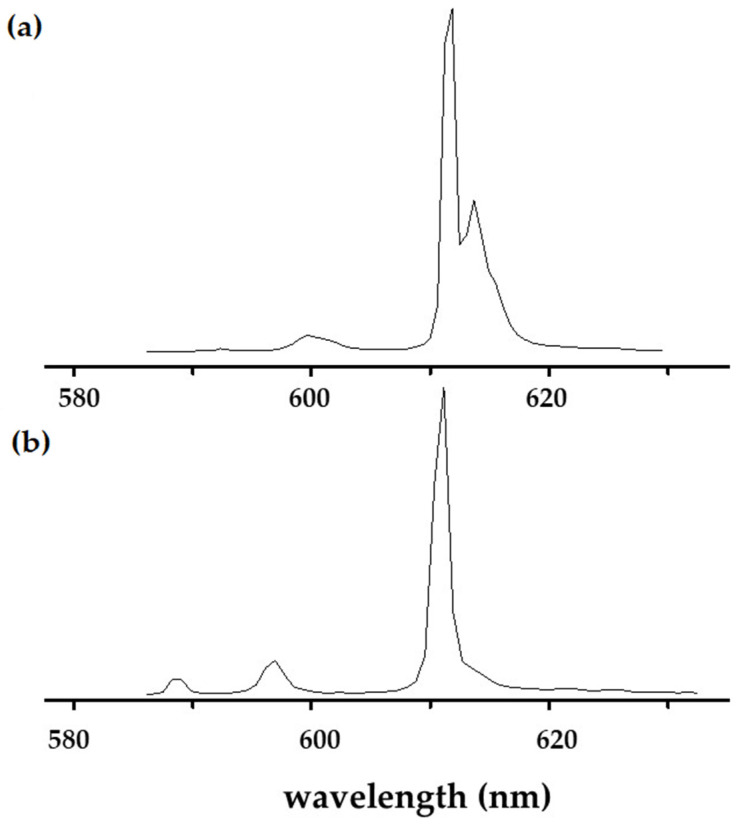
Normalized emission spectra of (**a**) **1** and (**b**) [Chol][Eu(fod)_4_], relative to the most intense ^5^D_0_→^7^F_2_ transition band at 30 °C in the solid state.

It was previously reported that the cations of [Eu(β-diketonate)_4_]^−^ salts can have a strong influence on the structure of the anion and consequently on the optical properties of the complexes [23,24]. In this case, despite having the same coordination sphere around the Eu(III), the luminescence properties of **1** and [Chol][Eu(fod)_4_] are significantly different (Table 1) in terms of absolute emission quantum yield (Φ, %), emission lifetime and ^5^D_0_→^7^F_2_ transition band splitting (Figure 2).

Higher lifetime of the ^5^D_0_ excited state (Table 1) is an indication of poorly efficient nonradiative relaxation processes due to the good shielding of the Eu (III), which is related, in this case, not only with the nature of the β-diketonate ligand, tetrakis-1,1,1,2,2,3,3-heptafluoro-7,7-dimethyloctane-4,6-dionate (fod), but also with the nature of the counter-ion.

Assuming residual anion-cation interactions at room temperature, an absolute quantum yield of 100% for **1** could be explained by the absence of any cation interaction with the “fod” ligands which is responsible for the antenna effect that increases the luminescence efficiency of Eu(III) center.

The emission profiles were measured at excitation wavelengths obtained from their corresponding excitation spectra, 345 nm for **1** and 340 nm for [Chol][Eu(fod)_4_].

Figure 2 presents the emission spectra recorded for **1** and [Chol][Eu(fod)_4_] in the solid state, in the 575–630 nm spectral range, corresponding to the ^5^D_0_→^7^F_J_ (J = 1–2) transitions of Eu(III).

The most intense ^5^D_0_→^7^F_J_ transition is an electric dipole transition and its intensity and splitting are very sensitive to the local symmetry and the nature of the co-sensitizers.

The intensity ratio R_21_, also known as the asymmetry ratio, indicates the degree of distortion from the inversion symmetry of the local environment around the Eu(III) ions [26]. This fact means that the nature of the cations of the presented salts strongly affects the local environment since to a higher asymmetry parameter the luminescent center is located more apart from a centrosymmetric geometry.

To compare the luminescence intensities of **1** and [Chol][Eu(fod)_4_], the ratio (R_21_) between the area under the emission curve of the hypersensitive ^5^D_0_→^7^F_2_ and of the magnetic dipole allowed ^5^D_0_→^7^F_1_ were determined. For **1** we found a value for I(^5^D_0_→^7^F_2_)/I(^5^D_0_→^7^F_1_) of 15.5 and for the [Chol][Eu(fod)_4_] a much lower R_21_ value of 5.6; to a higher ratio corresponds a more intense red emission. These R_21_ values are much higher than the value 0.67 of a centrosymmetric europium (III) complex [27], meaning that the Eu(III) ion is in a low site symmetry without an inversion center in the anionic [Eu(fod)_4_]^−^ core.

As can be observed, the ^5^D_0_→^7^F_2_ line of [Chol][Eu(fod)_4_] is much narrower than the majority obtained for other complexes, reflecting, as was said previously a I(^5^D_0_→^7^F_2_)/I(^5^D_0_→^7^F_1_) ratio of 5.6.

#### 3.1.2. Temperature Effect

We referred above that [Chol][Eu(fod)_4_] [25] presented an unusual thermochromic behavior, similar to what was previously reported for the ionic liquid [P_6,6,6,14_][Eu(fod)_4_] [14] which changed from light yellow to deep red around 80 °C.

**Figure 3:**
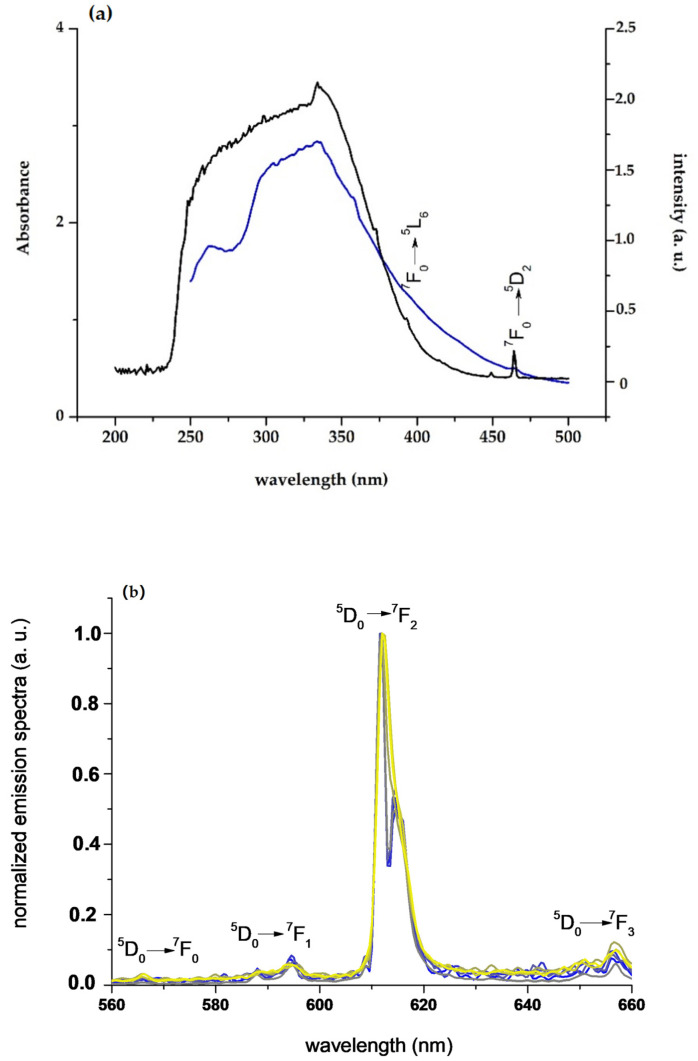
(**a**) Absorption spectra of **1** (blue line) and excitation spectra (black line) monitored with λ_em_ = 612 nm. (**b**) Normalized emission spectra of **1** relative to the maximum of ^5^D_0_→^7^F_2_ transition band (25 °C), from 25 (blue line) to 55 °C (yellow line) with a 5 °C·min^−1^ step size between each measurement with λ_exc._ = 345 nm.

Figure 3a displays the absorption and the excitation spectra of **1** acquired at ambient temperature monitoring the emission at their corresponding maxima of 612 nm.

Both spectra display a large broad band ascribed to the excited states of the ligands (260–450 nm), with one small peak around 393 [28]. A line at ca. 465 nm is assigned to the ^7^F_0_→^5^D_2_ Eu (III) intra-4f^6^ transition and is observable in both the absorption and excitation spectra. 

**Figure 4:**
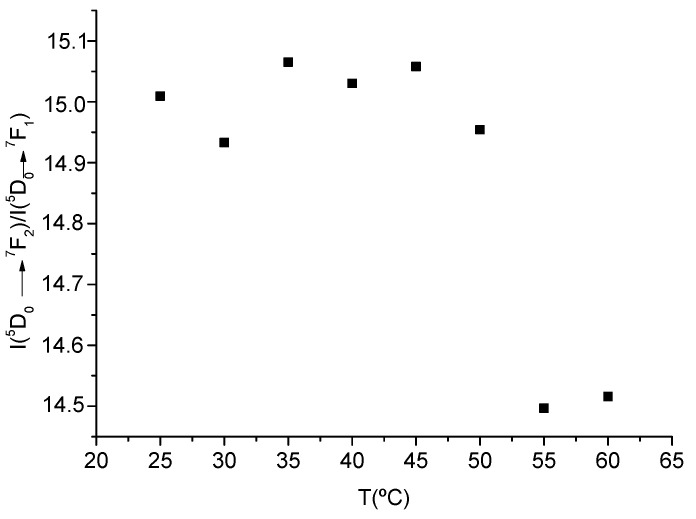
Temperature dependent total integrated intensity of ^5^D_0_→^7^F_2_ transition relative to the ^5^D_0_→^7^F_1_ transition band between 25 and 55 °C.

The ^5^D_0_→^7^F_0_ is a forbidden transition, showing very week emission. The ^5^D_0_→^7^F_1_ peak splitting was maintained unaltered during all temperature range (Figure 3b). This transition is a magnetic dipole (MD) transition and as such is largely independent of the environment of the Eu (III) ion. The total intensity of this transition was then used to calibrate the intensity of the luminescence spectra at different temperatures (Figure 4).

For **1** it was not observed any thermochromism in the visible range and upon heating the emission spectra profile was maintained constant until it starts to melt at temperatures close to 60 °C (Figure 3b). Even though, the emission spectra of **1** was monitored from room temperature, with a 5 °C degree step between 25 and 60 °C (Figure 4).

Complex **1** melt at 60 °C and no ratiometric relations were found with temperature increase. A low melting is most likely a result of the aliphatic long chain which also reduces the cation–anion interactions, possibly due to steric effects. 

The ^5^D_0_ decay curves of the Eu (III), irrespective of the emission line detected, the excitation wavelength selected and the temperature, can always be fitted by a single exponential function giving lifetime values 0.63 ± 0.01 ms, for **1** (Figure S5).

The thermal properties of **1** and **1/PSU** were examined using differential scanning calorimetry (DSC) and thermogravimetry (TG) on powder samples. By the TG analysis (Figure S3), compound **1** is stable until close to 170 °C, while the PSU matrix only starts to degrade after 450 °C. By the DSC analysis (Figure S4), the melting of **1** occurs at 59.5 °C (with an onset at 53.3 °C and an offset at 62.7 °C) and this transition is visible in 1/PSU as a “flattened” region in the DSC curve.

### 3.2. Characterization of 1/PSU and [Chol][Eu(fod)_4_]/PSU with 10% (w/w) of the Complex

**Figure 5:**
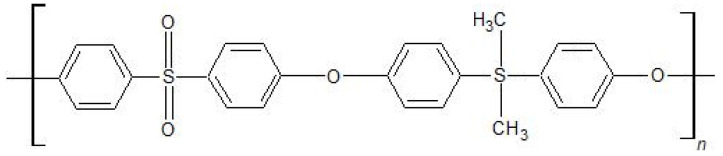
Schematic representation of the PSU polymer.

Here, we summarize the spectral evidence of Eu (III) energy transfer in PSU hybrids incorporating both **1** and **[Chol][Eu(fod)_4_]** in 10% (*w*/*w*) in polysulfone. The schematic representation of the PSU polymer is presented in Figure 5. Preparation, photophysical and thermochromic properties of these functionalized materials were never reported before.

#### 3.2.1. Photophysical Characterization

The luminescence decay lifetimes (τ) and the quantum efficiency (Φ) for the hybrid materials were also evaluated as a measure of the efficiency of the emission process (Table 2). The luminescence lifetimes (τ) of the film with 10% of Eu (III) complex is 0.63 ms, identical to what was found for **1** (Figures S5 and S6). According with Figures S5 and S6, both the hybrid film and the free complex exhibited a single exponential decay with the same lifetime value suggesting the same radiative decay pathway within the imbibed molecule. The absorption and excitation spectra of pristine PSU is presented in Figure S7.

#### 3.2.2. Thermochromism with Temperature-Responsive Ratiometric Behavior of 1/PSU and [Chol][Eu(fod)_4_]/PSU 

**Figure 6:**
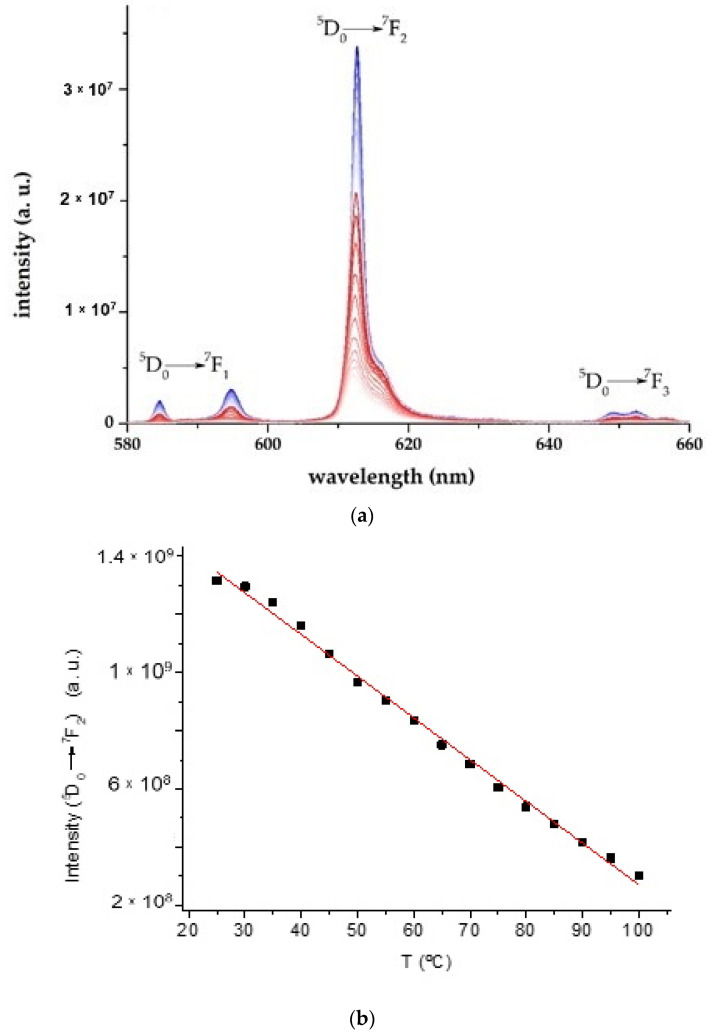
(**a**) emission spectra of **1/PSU** at different temperatures upon excitation at 320 nm. Most intense blue line at room temperature, less intense red line acquisition ate 135 °C; (**b**) total intensity of ^5^D_0_→^7^F_2_ transition band between 25 and 100 °C with 5 °C step, y = −1.43x + 1.70 (r^2^ = 0.99).

Similar to the observed for **1**, the doped membrane **1/PSU** presents a reduction in the emission intensity of Eu (III) that is not accompanied by any significant changes in its profile (Figure 6a). **1/PSU** film presented a usually observed and commonly reported temperature-dependent luminescence intensity with a linear fit from 60 to 100 °C (Figure 6b).

**Figure 7:**
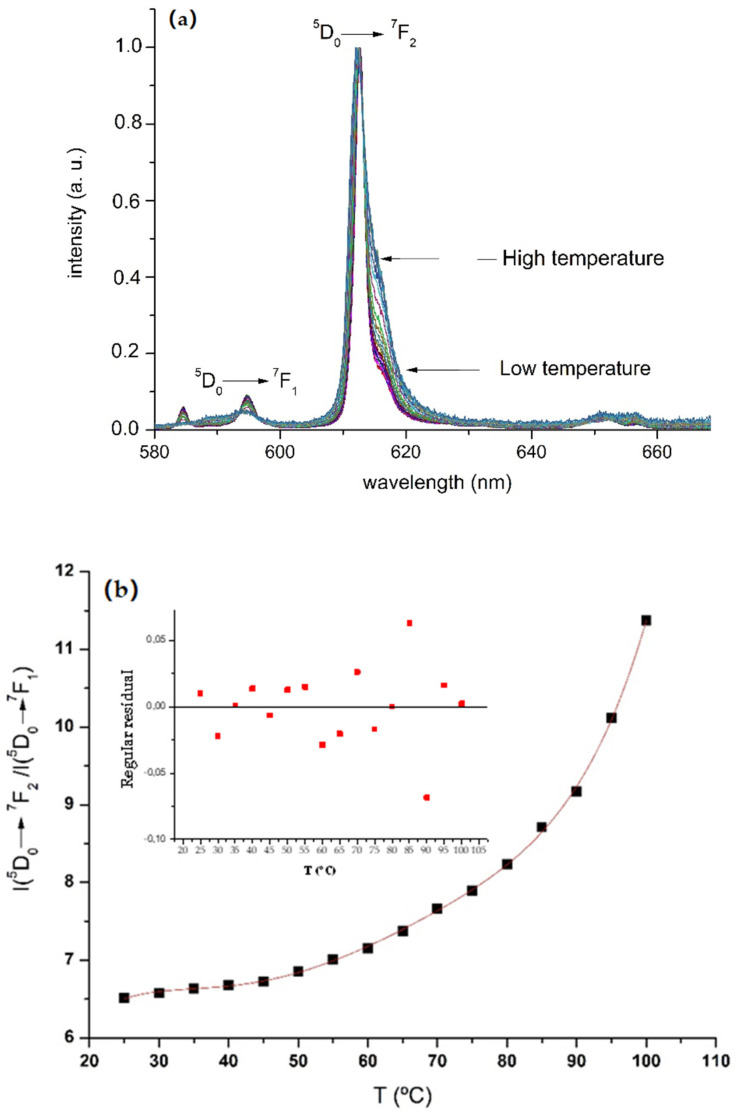
(**a**) emission spectra of [**Chol][Eu(fod)_4_]/PSU** at different temperatures (from 25 to 160 °C) with λ_exc_ = 325 nm; (**b**) Thermochromic response of [**Chol][Eu(fod)_4_]/PSU** film from 25 to 100 °C with polynomial fit y = 0.875 + 0.597x – 0.024x^2^ + 4.66 × 10^−4^x^3^, r^2^ = 0.99. Inset: regular residual associated with each measurement.

In Figure 7a we present the emission spectra of **[Chol][Eu(fod)_4_]/PSU** with 10% loading of Eu (III) complex when exposed at temperatures between 25 and 160 °C. The feature that allows the ratiometric relation found and presented in Figure 7b is the relative increase of the intensity of the shoulder at 616 nm, from low to higher intensities as the temperature increases. Figure 6b represents the relations found between the total integrated intensity of the ^5^D_0_→^7^F_2_ relative to the total intensity of the ^5^D_0_→^7^F_1_ magnetic transition for the **[Chol][Eu(fod)_4_]/PSU** membrane. A polynomial fit can be used to relate ratiometrically the emission bands intensity with temperature, with the equation y = 0.875 + 0.597x – 0.024x^2^ + 4.66 × 10^−4^x^3^. Above 100 °C we did not consider the ratiometric relation due to a random variation of the ratio, eventually caused by reduction of chemical stability of the Eu (III) complex.

#### 3.2.3. Images of 1/PSU and [Chol][Eu(fod)_4_]/PSU 10% (*w*/*w*)

**Figure 8:**
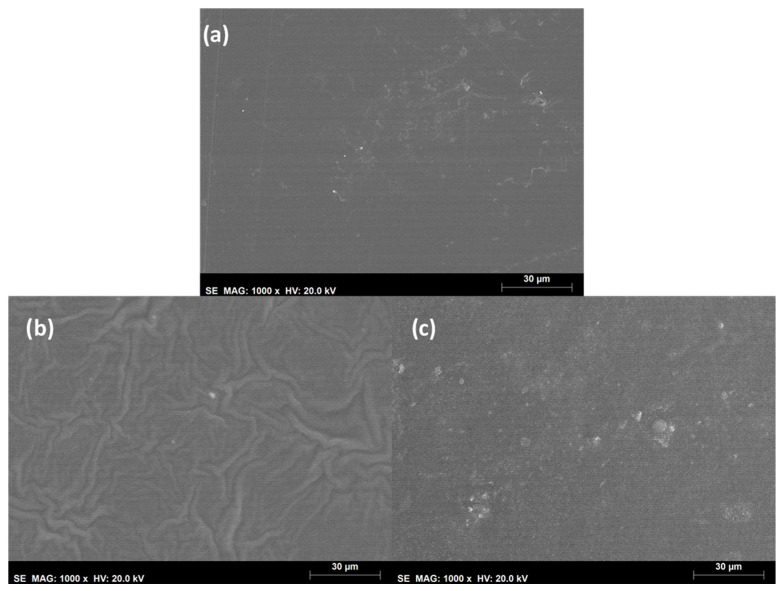
(**a**) SEM image of top surface **PSU** with 1000× magnification. (**b**) SEM image of top surface of **1/PSU** (10% *w*/*w*) nanocomposite with 1000× magnification showing micelles formed by **1**. (**c**) SEM image of top surface of **[Chol][Eu(fod)_4_]/PSU** (10% *w*/*w*) nanocomposite with 1000× magnification.

N-cetylpyridinium chloride, C_16_PyrCl, is classified as a cationic surfactant, and the long structures visible in the SEM image of **1/PSU** (Figure 8b) are attributed to the formation of micelles [29]. Despite the long C_16_ aliphatic chains the hydrophilic character of **1/PSU** film is higher than found for [Chol][Eu(fod)_4_]/PSU as was confirmed by the results obtained from the measurements of the water contact angles (Table 3). Lower contact angles correspond to good adhesiveness, good wettability, and high solid surface free energy.

Confining 10% of **1** or [Chol][Eu(fod)_4_] induced, according with the commonly known experience [30], increase of the water contact angle of the hybrid membrane can be attributed to the hydrophobic nature of the guest material.

Sanz-Medel and co-workers also suggested that both electrostatic and hydrophobic interactions concurrently promote fluorescence yield in micellar solutions [31].

**Figure 9:**
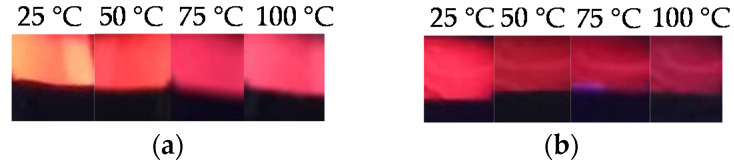
Images of (**a**) 1/PSU and (**b**) [Chol][Eu(fod)_4_]/PSU under 366 nm UV lamp at different temperatures.

Figure 9 shows the color of the studied membranes under UV light at 25, 50, 75 and 100 °C.

## 4. Discussion

The experimented cations induced significant fluorescence changes in the emissive specie which provide different sensing possibilities performed by the anionic [Eu(fod)_4_]^−^ core. Specific properties of the cholinium cation, that allow thermochromism in the solid state in combination with the [Eu(fod)_4_]^−^ anion, are not present in the N-cetylpyridinium as a counter-ion. Confinement in a polymer film of polysulfone maintains the presence of a ratiometric behavior although the linearity previously observed for the [Chol][Eu(fod)_4_] is lost and instead a polynomial relation is established in the emissive film.

## 5. Conclusions

The experimented cations induced significant fluorescence changes in the emissive specie which may provide several sensing possibilities performed by the anionic [Eu(fod)_4_]^−^ core which can be preserved after confinement in a polysulfone matrix. In opposition to the observed previously, higher amount of Eu (III) complex allows establishing a relation between temperature and the ratio between two transition bands of a thin film of [Chol][Eu(fod)_4_]/PSU. The unique thermochromic behavior discussed here deserves further studies in the near future and we intend to perform further work using different materials for biological applications.

## Figures and Tables

**Table 1:** Emission lifetime (τ, ms) and absolute emission quantum yield (Φ, %) of **1** and [Chol][Eu(fod)_4_].

-	1	[Chol][Eu(fod)_4_] [25]
τ (ms)	0.63	1.26
Φ (%)	100	81

**Table 2:** Emission lifetime (τ, ms, Figures S5 and S6) and absolute emission quantum yield (Φ, %, Figure S8) of **1** and **[Chol][Eu(fod)_4_]** and confined polysulfone films with 10% loading.

-	1	1/PSU	[Chol][Eu(fod)_4_] [25]	[Chol][Eu(fod)_4_]/PSU
τ (ms)	0.63	0.62	1.26	0.87
Φ (%)	100	21	81	24

**Table 3:** Water contact angles of pristine PSU and modified PSU membranes with 10% of **1** and [Chol][Eu(fod)_4_].

**-**	**PSU**	**1/PSU**	**[Chol][Eu(fod)_4_]/PSU**
Water contact angle (°)	66	79	84
	-	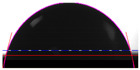	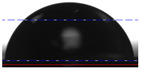

## Supplementary Materials

The following are available online at https://www.mdpi.com/1996-1944/13/23/5394/s1, Figure S1: ESI-MS analysis of compound 1 in acetonitrile, positive mode, Figure S2. ESI-MS analysis of compound 1 in acetonitrile, negative mode, Figure S3: Thermogravimetric profile of **PSU** (green), **1-PSU** (blue) and **1** (red), Figure S4: Differential Scanning Calorimetry analysis of **1** (red) and **1/PSU** (blue), Figure S5. Luminescence decay curves of **1** at emission maxima of 612 nm, Figure S6: Luminescence decay curves of **1/PSU** at emission maxima of 612 nm, Figure S7. Absorption (up) and excitation (down) spectra of pristine PSU, Figure S8. Schematic representation of the three-measurement approach; L (up) refers to excitation and P (down) to emission: La empty sphere, Lb sample out of the beam, Lc sample in the path of the incident beam.
