# Supplementary material for: Thermochromism of Highly Luminescent Photopolymer Flexible Films Based On Eu (III) Salts Confined in Polysulfone
Source: Materials (Basel). 2020 Nov 27;13(23):5394. doi: 10.3390/ma13235394 (PMC7729765; doi:10.3390/ma13235394)
Supplement: Supplementary file 1 [file materials-13-05394-s001.pdf]

materials

Thermochromism of Highly Luminescent Photopolymer Flexible Films based on Eu (III) Salts Confined in Polysulfone

Mani Outis [1], João Paulo Leal [2], Maria Helena Casimiro [3], Bernardo Monteiro [4]* and Cláudia Cristina Lage Pereira [1,]*

[1] LAQV-REQUIMTE, Departamento de Química, Universidade Nova de Lisboa, 2829-516 Caparica, Portugal.; m.hosseinzadeh@campus.fct.unl.pt , ccl.pereira@fct.unl.pt
[2] Centro de Química Estrutural (CQE), DECN, Instituto Superior Técnico, Universidade de Lisboa, Estrada Nacional 10, 2695-066 Bobadela, Portugal.; jpleal@ctn.tecnico.ulisboa.pt
[3] Centro de Ciências e Tecnologias Nucleares (C²TN), Instituto Superior Técnico, Universidade de Lisboa, Estrada Nacional 10, 2695-066 Bobadela, Portugal.; casimiro@ctn.tecnico.ulisboa.pt
[4] Centro de Química Estrutural (CQE), DEQ, Instituto Superior Técnico, Universidade de Lisboa, Estrada Nacional 10, 2695-066 Bobadela, Portugal.; bernardo.monteiro@ctn.tecnico.ulisboa.pt
* Correspondence: bernardo.monteiro@ctn.tecnico.ulisboa.pt (B.M.); ccl.pereira@fct.unl.pt (C.C.L.P.)

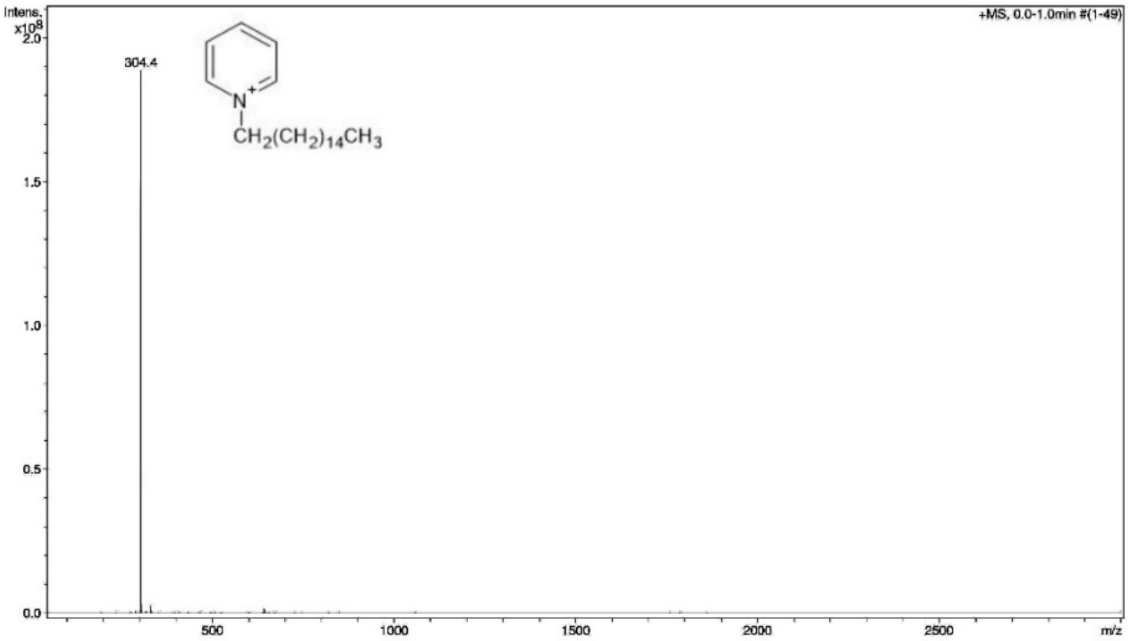

Figure S1. ESI-MS analysis of compound 1 in acetonitrile, positive mode.

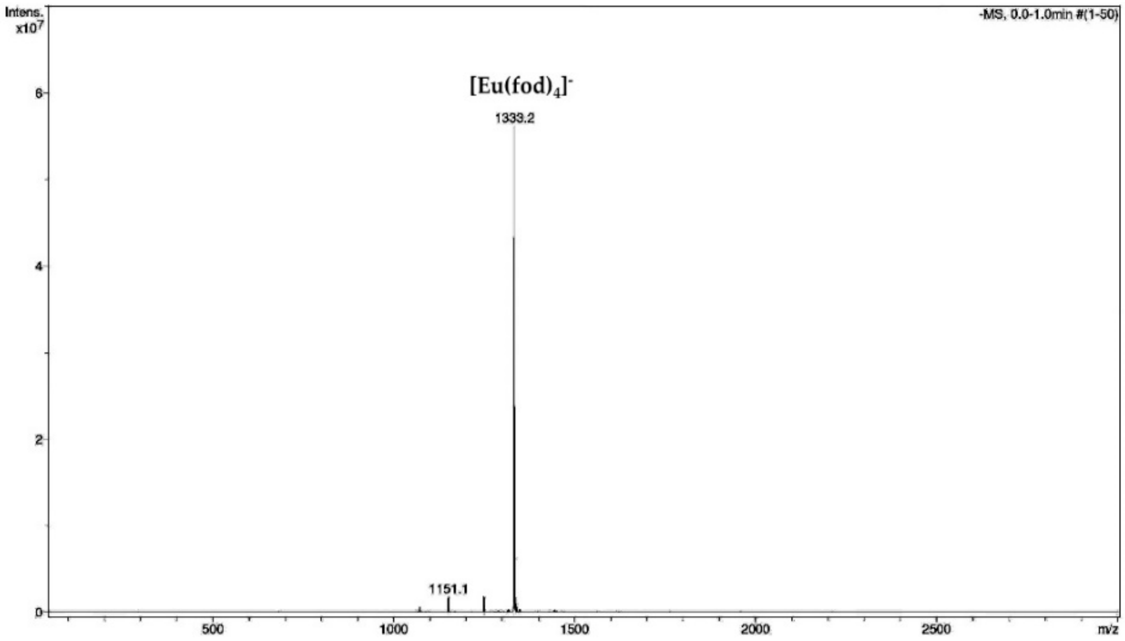

Figure S2. ESI-MS analysis of compound 1 in acetonitrile, negative mode.

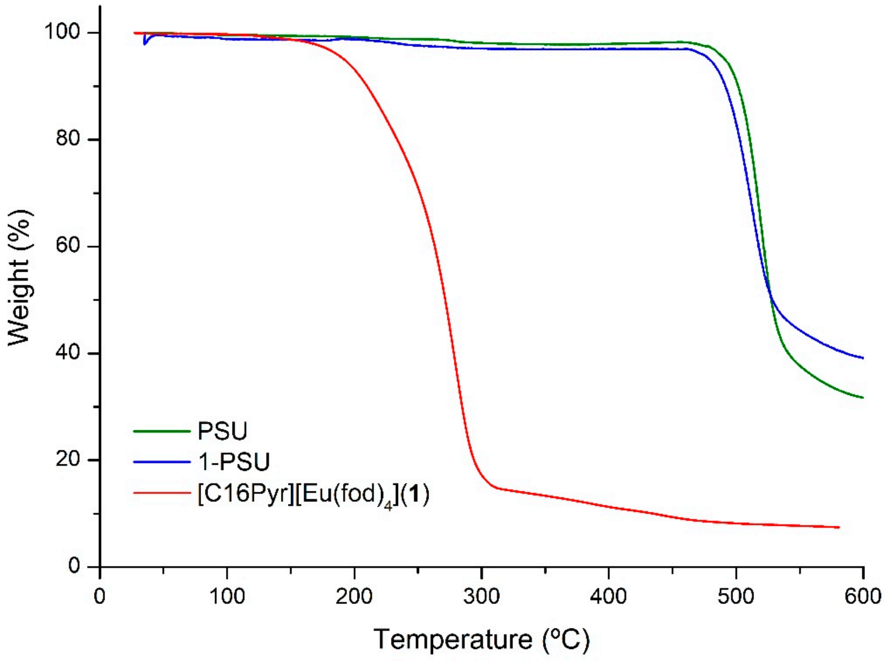

Figure S3. Thermogravimetric profile of **PSU** (green), **1-PSU** (blue) and **1** (red).

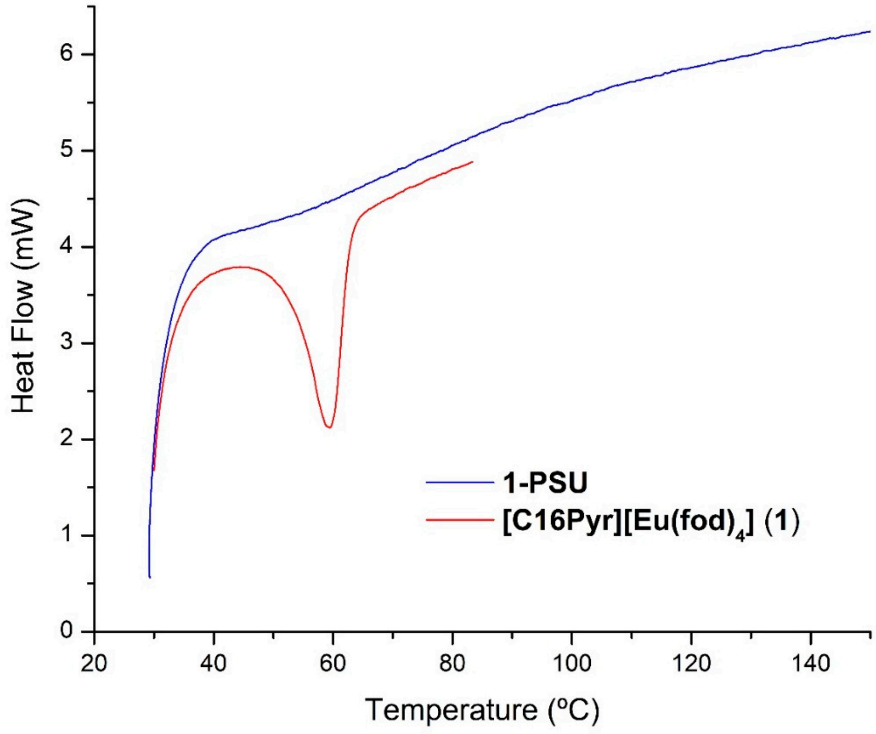

Figure S4. Differential Scanning Calorimetry analysis of **1** (red) and **1/PSU** (blue).

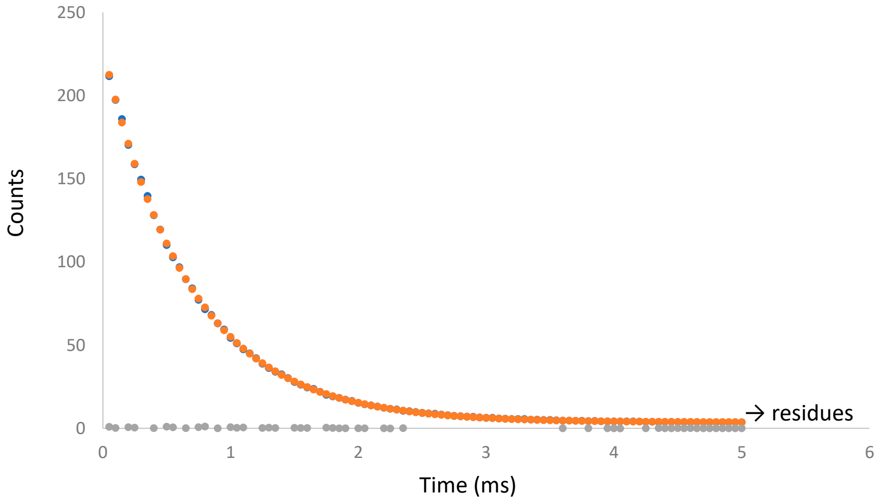

Figure S5. Luminescence decay curves of **1** at emission maxima of 612 nm.

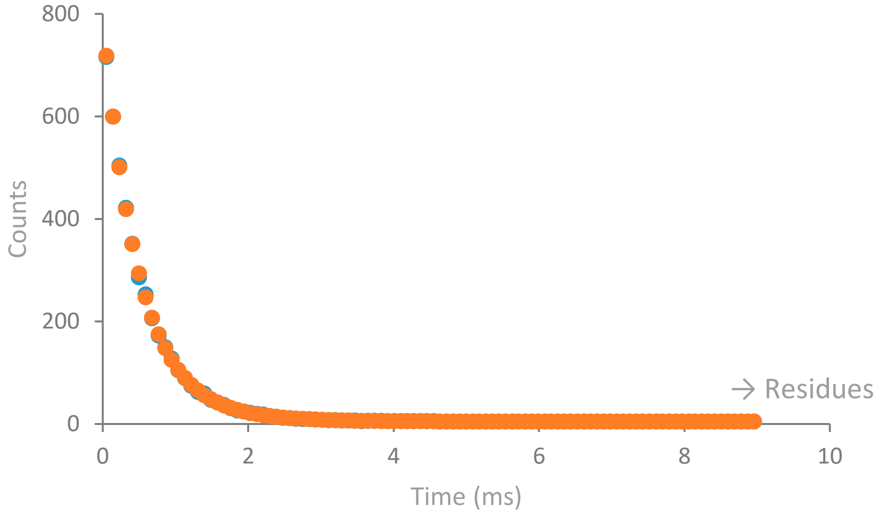

Figure S6. Luminescence decay curves of **1/PSU** at emission maxima of 612 nm.

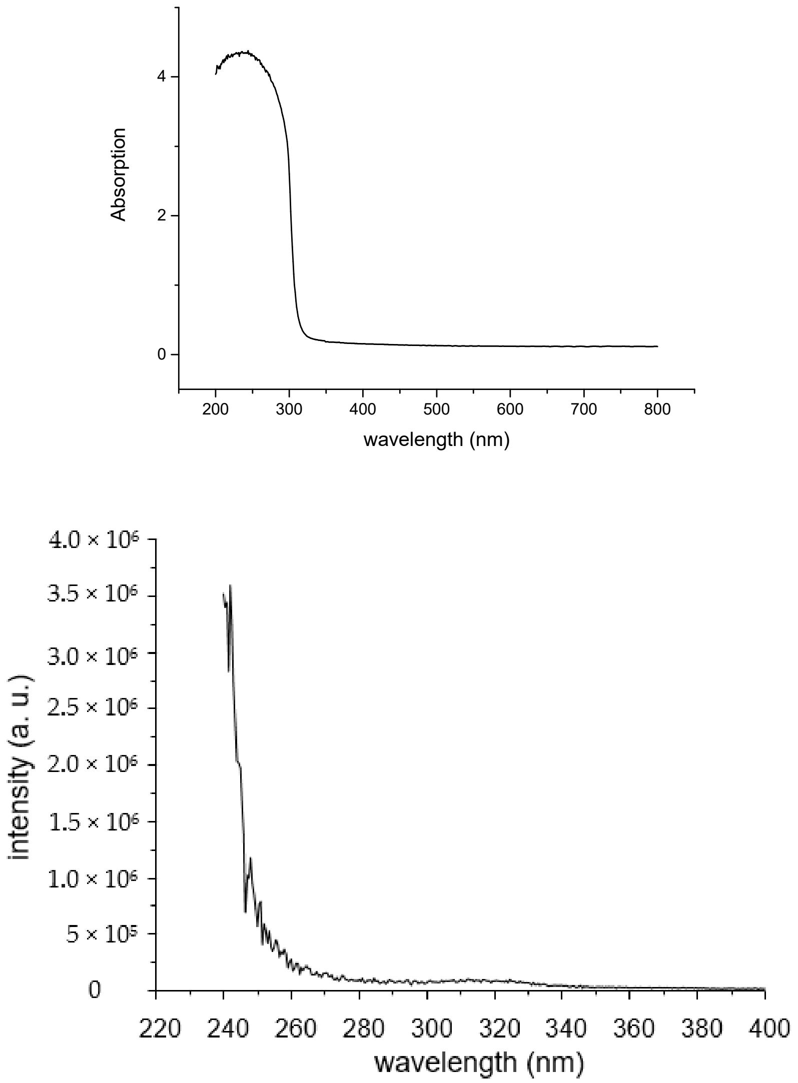

Figure S7. PSU absorption (up) and excitation (down) spectra of pristine PSU.

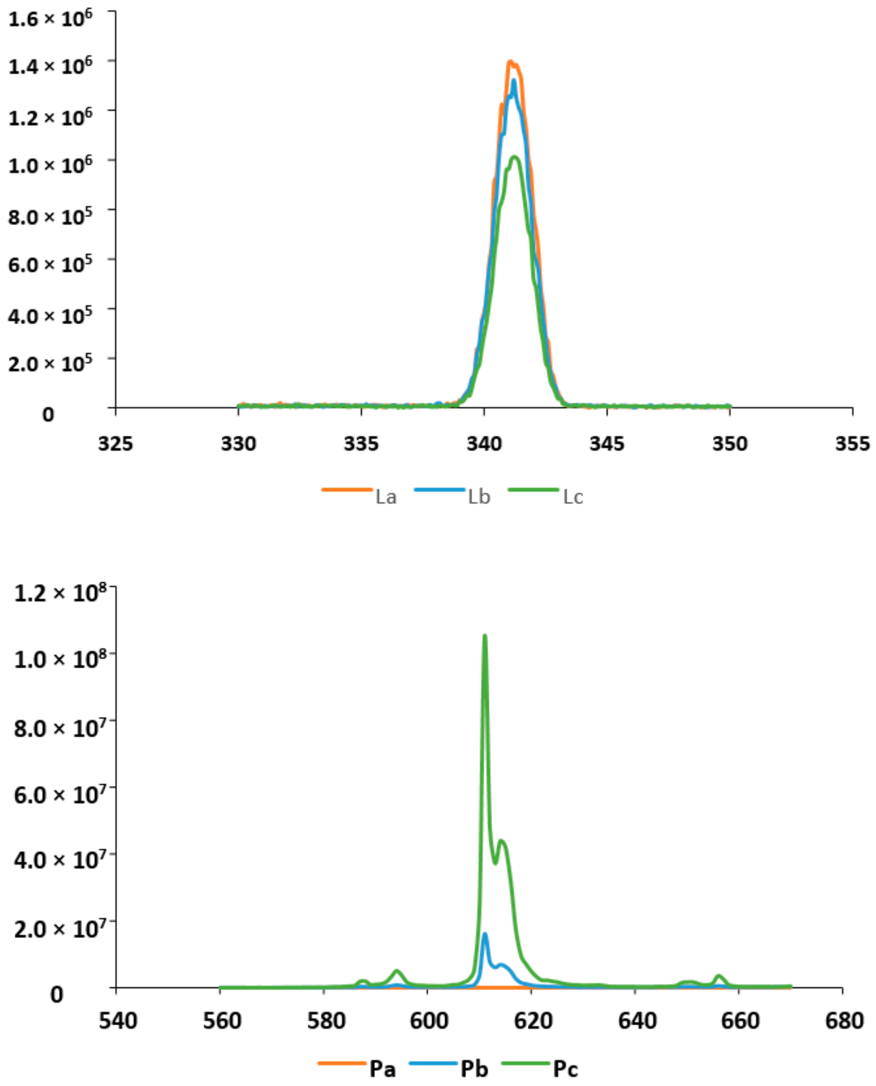

Figure S8. Schematic representation of the three-measurement approach; L (up) refers to excitation and P (down) to emission: La empty sphere, Lb sample out of the beam, Lc sample in the path of the incident beam.

Publisher's Note: MDPI stays neutral with regard to jurisdictional claims in published maps and institutional affiliations.

© 2020 by the authors. Licensee MDPI, Basel, Switzerland. This article is an open access article distributed under the terms and conditions of the Creative Commons Attribution (CC BY) license (http://creativecommons.org/licenses/by/4.0/).
